# YOLOv8-LBP: multi-scale attention enhanced YOLOv8 for ripe tomato detection and harvesting keypoint localization

**DOI:** 10.3389/fpls.2025.1656381

**Published:** 2025-09-11

**Authors:** Wang Yong, Xu Shunfa, Cheng Konghao

**Affiliations:** School of Computer Science and Technology, Zhejiang University of Technology, Hangzhou, China

**Keywords:** ripe tomato recognition, keypoint prediction, YOLOv8-Pose, attention mechanism, multi-scale feature fusion

## Abstract

In the process of target detection for tomato harvesting robots, there are two primary challenges. First, most existing tomato harvesting robots are limited to fruit detection and recognition, lacking the capability to locate harvesting keypoints. As a result, they cannot be directly applied to the harvesting of ripe tomatoes. Second, variations in lighting conditions in natural environments, occlusions between tomatoes, and missegmentation caused by similar fruit colors often lead to keypoint localization errors during harvesting. To address these issues, we propose YOLOv8-LBP, an enhanced model based on YOLOv8-Pose, designed for both ripe tomato recognition and harvesting keypoint detection. Specifically, we introduce a Large Separable Kernel Attention (LSKA) module into the backbone network, which effectively decomposes large kernel convolutions to extract target feature matrices more efficiently, enhancing the model’s adaptability and accuracy for multi-scale objects. Secondly, the weighted bidirectional feature pyramid network (BiFPN) introduces additional weights to learn the importance of different input features. Through top-down and bottom-up bidirectional paths, the model repeatedly fuses multi-scale features, thereby enhancing its ability to detect objects at multiple scales. Ablation experiments demonstrate that, on our self-constructed ripe tomato dataset, the YOLOv8-LBP model achieves improvements of 4.5% in Precision (*P*), 1.1% in *mAP*_50_, 2.8% in *mAP*_50−95_, and 3.3% in *mAP*_50−95_ − *kp* compared to the baseline. When compared with the state-of-the-art YOLOv12-Pose, YOLOv8-LBP shows respective improvements of 5.7%, 0.5%, 3.5%, and 4.9% in the same metrics. While maintaining the improvement in model accuracy, our method introduces only a small computational overhead, with the number of parameters increasing from 3.08M to 3.175M, GFLOPs rising by 0.1, and the inference speed improving from 96.15 FPS to 99.01 FPS. This computational cost is reasonable and acceptable. Overall, the proposed YOLOv8-LBP model demonstrates significant advantages in recognizing ripe tomatoes and detecting harvesting keypoints under complex scenarios, offering a solid theoretical foundation for the advancement of robotic harvesting technologies.

## 1 Introduction

Smart agriculture is an emerging trend in the modernization of agricultural practices, playing an indispensable role in the high-quality development of agriculture in China. The vigorous development of smart agriculture has become a key driving force in achieving rural revitalization Yu and Haiyan (2025). As an important tool of agricultural intelligence, harvesting robots are capable of performing efficient and precise picking operations, significantly reducing labor costs while improving production efficiency Zhang et al. (2023).

At the core of harvesting robots lies the target detection algorithm, which provides accurate target localization guidance for the robot’s hydraulic control system Zhang et al. (2024b). Cai et al. (2024) proposed a cherry tomato detection method based on multimodal perception and an improved YOLOv7-tiny network. By utilizing RGB-D image inputs and introducing a “Classness” prediction mechanism along with a hybrid non-maximum suppression strategy, the method enhances detection accuracy and real-time performance. Experimental results demonstrate that the method achieves a high picking success rate and exhibits promising potential for practical applications in greenhouse environments. Liang et al. (2025) developed an improved CTDA model based on YOLOv8 by redesigning the backbone network and incorporating SoftPool and an attention-driven dynamic detection head to improve small object feature extraction and multi-scale feature fusion efficiency. Under complex harvesting conditions, the model achieved a detection accuracy of 94.3% and a *mAP*_50−95_ of 76.5%, with a detection speed of 154.1 FPS and a model size of only 6.7 MB, demonstrating excellent real-time performance and robustness. Wu et al. (2025) introduced a lightweight detection network, LEFF-YOLO, based on an improved YOLOv8 framework. They incorporated the VanillaNet module into the backbone to reduce computational cost and enhanced feature fusion capability using the SiMAM attention mechanism and the SiMAMC2f module. Furthermore, a novel bounding box loss function was designed to improve model stability, resulting in improvements of 2.7% and 2.9% in *mAP*_50_ and *mAP*_50−95_ respectively, with a 32% reduction in parameters and a 10.5% decrease in computational load, meeting the real-time and accuracy requirements of harvesting robots. Liu et al. (2024) proposed a “coarse detection–fine segmentation” method, Y-HRNet, tailored for greenhouse environments. The approach first employs YOLOv7 for lightweight multi-class tomato detection, and then utilizes a Y-HRNet network enhanced with ECA and DR-ASPP modules for pixel-level segmentation of green, turning, ripe, and fully ripe tomatoes. This method achieved a mean IoU of 84.69% and an accuracy of 94.39% under complex backgrounds, with an average processing time of 0.35 seconds, providing strong support for tomato maturity grading and harvesting management. Zhang et al. proposed a tomato detection algorithm for harvesting robots based on Shufflenetv2-YOLOv5, utilizing Shufflenetv2 as the backbone to reduce computational cost, incorporating the ECA attention mechanism, and replacing the activation function with FReLU to enhance detection precision and model robustness Zhang et al. (2024a). However, the above approaches primarily focus on fruit-level object detection, without addressing keypoint localization for harvesting. As a result, although these methods can effectively detect fruits, they lack the capability for precise picking point localization, making them difficult to directly apply in real-world tomato harvesting operations.

In the field of tomato picking point localization, Liu Rong et al. achieved automatic harvesting by detecting tomato branches, including coarse localization of fruits and pixel-level segmentation of multiple pedicels Rong et al. (2021). However, their method exhibited a significant angular error of up to 5°. Luo et al. (2018) proposed a grape stem cutting point detection method based on geometric modeling and contour analysis, which integrates K-means clustering segmentation with a geometric constraint strategy. In complex vineyard environments, this approach achieved an average recognition accuracy of 88.33% and a double-cluster stem cutting point detection success rate of 81.66%, providing a feasible solution for the application of harvesting robots in operations involving overlapping grape clusters. Sa et al. utilized HSV color space and geometric features (such as curvature differences between stems and fruit surfaces), combined with a support vector machine (SVM) to extract features and predict the location of sweet pepper pedicels Sa et al. (2017).

These methods mainly rely on geometric features for predicting harvesting points. However, in practical operations, factors such as ambient lighting, fruit occlusion, and leaf interference can significantly impact accuracy, leading to misaligned or missed picking points, thereby increasing the risk of failed or inaccurate harvesting and limiting their application in complex environments.

Keypoint detection was originally applied in human pose estimation. For instance, JEONG et al. used the OpenPose algorithm to infer smoking behavior through skeleton graphs constructed from human keypoints Feng et al. (2024). Today, many studies have extended this technique to industrial and agricultural domains. For example, Taehyeong Kim et al. used OpenPose as the backbone to estimate the posture of tomato fruits Kim et al. (2023). Wu et al. proposed a stem localization method for grapes, based on their top-down growth characteristics, where grape clusters were detected using object detection and the stem positions were then accurately located through keypoint detection Wu et al. (2023). Kang et al. (2025) focused on greenhouse-grown melons and employed Region of Interest (RoI) to coarsely localize ripe fruits, followed by Human Pose Estimation (HPE) to estimate the posture of melon-fruit-peduncle pairs within the RoI. Their approach achieves high performance with improved real-time efficiency, even under limited training data.

However, most of these methods were developed under controlled conditions such as greenhouses, relying on stable crop postures and standardized cultivation patterns. In contrast, tomatoes grown in natural environments may exhibit a wide range of unpredictable postures (e.g., hanging, inclined, or covered) due to wind, gravity, and occlusion by leaves and branches. These factors significantly limit the generalizability and robustness of existing approaches, making them difficult to apply effectively in real-world natural field conditions where tomato postures are highly variable and uncertain.

Debapriya Maji was among the first to unify keypoint detection and object detection in the context of human pose estimation Maji et al. (2022). Zhang et al. built upon YOLO-Pose by proposing an algorithm that fuses pose estimation with object detection, achieving an accuracy of 90.14% in dense crowd scenes—21.56% higher than conventional YOLO-Pose Zhang and Xue (2024). Qin et al. developed PW-YOLO-Pose, which effectively addressed challenges in power operation scenarios, such as complex backgrounds, occlusions, small targets, and extreme viewpoints, thereby reducing keypoint loss and false detections Su et al. (2024). Liu et al. developed an improved YOLOv8-Pose model by introducing a Slim-neck module and a CBAM attention mechanism module to recognize red-ripe strawberries and detect peduncle keypoints. The improved YOLOv8-Pose achieved a *mAP_kp_* of 97.91% Mochen et al. (2023).

Building on these insights, this paper improves upon human pose estimation algorithms for predicting keypoints related to the harvesting of ripe tomatoes. By analyzing the geometric configuration and growth posture of tomatoes, we aim to accurately predict keypoints at the fruit pedicel and picking region, thus offering technical support for robotic tomato harvesting. The main contributions of this study are as follows:

1. An attention mechanism (LSKA) Lau et al. (2024) is incorporated into the backbone network to effectively decompose large convolutional kernels, extract richer target feature matrices, and enhance the network’s feature extraction capability.
2. A Bidirectional Feature Pyramid Network (BiFPN) Lin et al. (2017) is introduced in the neck of the model to facilitate adaptive weighting during training and enable bidirectional information flow between high- and low-level features.
3. The YOLOv8-LBP model, when applied to our self-constructed ripe tomato dataset, achieves performance gains of +4.5% in *P*, +1.1% in *mAP*_50_, +2.8% in *mAP*_50−95_, and +3.3% in *mAP*_50−95_ − *kp*, demonstrating the effectiveness of the proposed improvements.

## 2 Material and methods

### 2.1 Material

#### 2.1.1 Self-constructed ripe tomato dataset

The dataset used in this study is derived from TomatoDiverse: An Open Dataset for Industrial Tomato Detection in Complex Natural Environments Tao (2024). The images were captured at tomato production bases located in Bayingolin Mongol Autonomous Prefecture, Xinjiang Uygur Autonomous Region, and Jingxian County, China. From this dataset, a total of 236 high-resolution images (4000×3000 pixels) depicting the growth posture of ripe tomatoes were selected.

**Figure 1:**
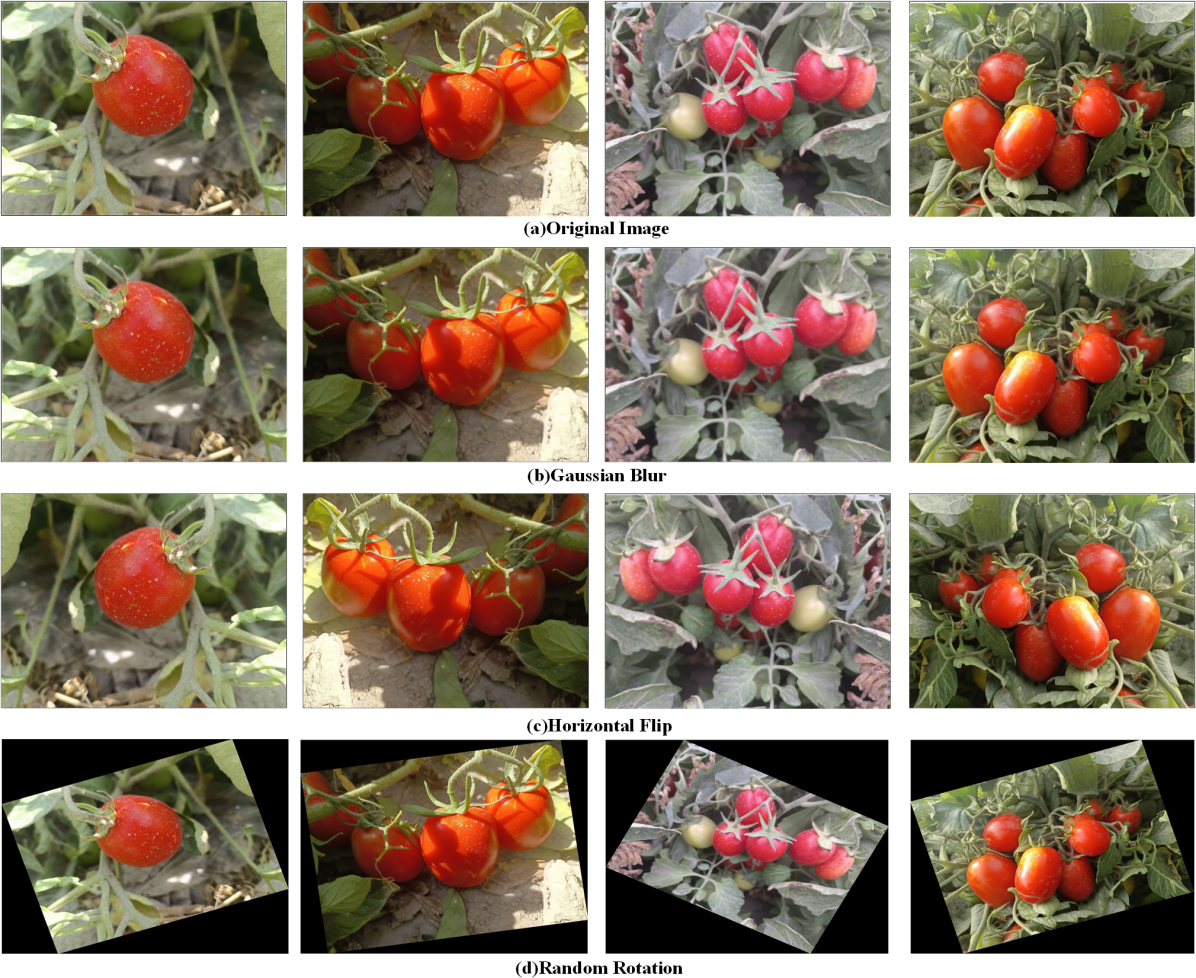
Ripe tomato images after data augmentation.

These images reflect a variety of challenges present in complex natural environments, including variations in lighting conditions, different shooting distances, and occlusions. To enhance data diversity, the dataset was augmented using several techniques: random rotation (with angles randomly sampled within the range from -30 to +30) Cubuk et al. (2019), horizontal flipping Krizhevsky et al. (2012), and Gaussian blurring (with Gaussian kernel sizes randomly selected from [21, 31, 41, 51]) Zhang et al. (2019). After augmentation, the dataset was expanded to a total of 780 images. **Figure 1** illustrates examples of the augmented images.

The criteria for determining red-ripe Choi et al. (1995) tomatoes in the dataset are as follows: 1. Visual Inspection: A tomato is labeled as ripe when the red area on its surface reaches or exceeds 90%. 2. Quantitative HSV-Based Determination: For samples where manual judgment is challenging, the Labelme tool is used to segment the tomato region, and the average values of Hue, Saturation, and Value (Brightness) are calculated in the HSV color space. A tomato is considered red-ripe if the following conditions are met: Hue (H): Within the range of 0 – 11.2 or 144 – 180 (where 11.2 is derived from dividing the visual threshold 10 by 0.9, and 144 is calculated similarly);Saturation (S): Between 100 and 255;Value (V): Between 100 and 255.

Mochen et al. (2023) adopted two keypoints (the peduncle and the picking point) to annotate and detect strawberries grown in an elevated cultivation system. In this cultivation mode, strawberry fruits hang naturally and maintain a stable posture, making keypoint annotation relatively easy. In contrast, tomatoes in natural growing environments may either hang down or lie horizontally on branches and leaves, exhibiting a wide variety of postures. To enable more effective localization of picking points, we introduce three keypoints in this study: P1 (picking point), P2 (peduncle location), and P3 (fruit bottom). P2 and P3 together provide information about the size and orientation of the fruit, which is critical for evaluating the feasibility of grasping. P1 is located approximately 1 – 2 cm above P2 along the direction of the peduncle and represents the ideal cutting point for the harvesting robot. This design ensures accurate picking without damaging the fruit itself and minimizes the risk of mistakenly cutting non-target parts such as branches or leaves.

**Figure 2:**
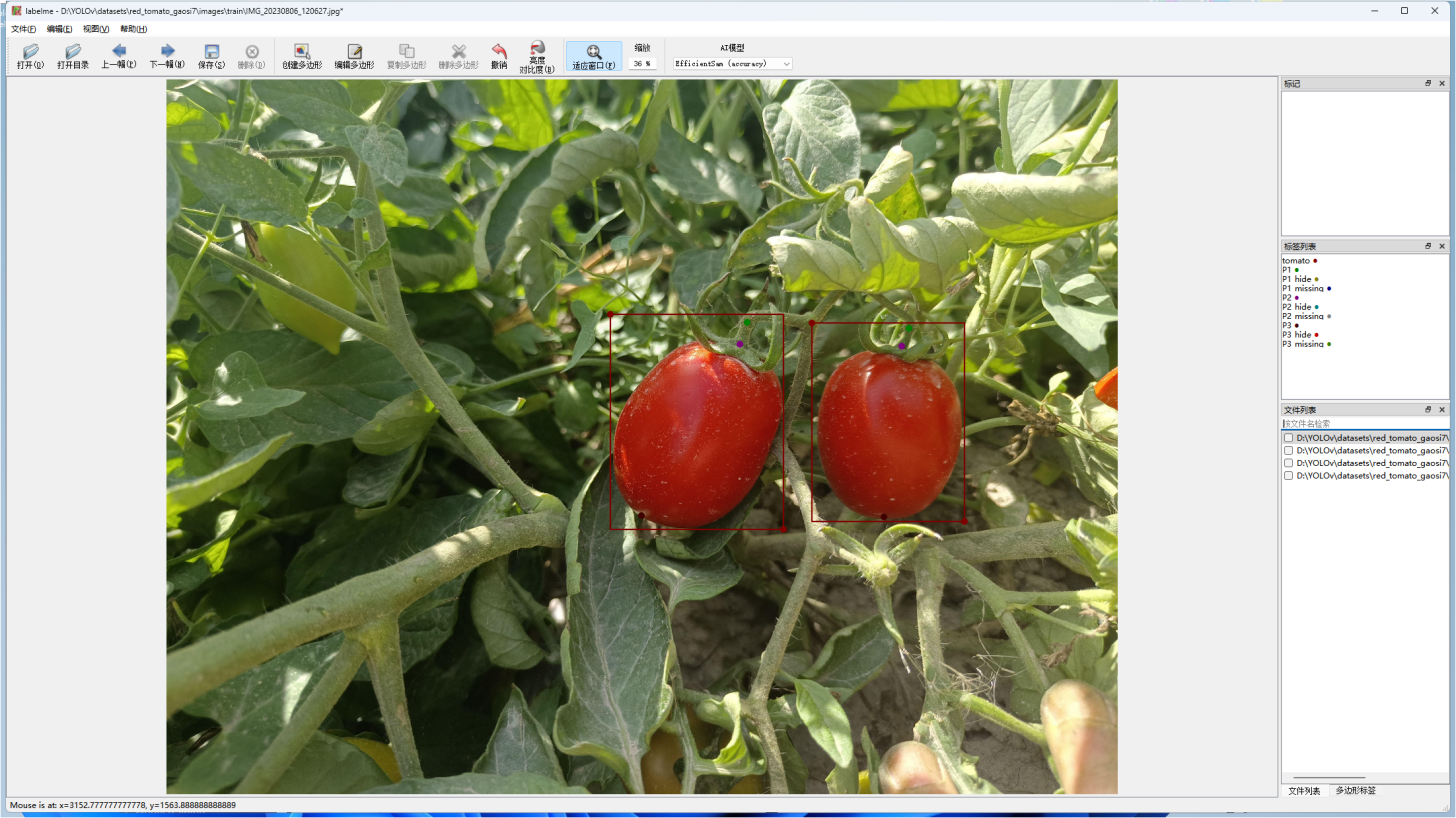
Annotation of red-ripe tomatoes.

Keypoint annotations for ripe tomatoes were performed using the Labelme software, with the tomato fruit and its picking points defined as annotation targets. Each ripe tomato was annotated using its minimum bounding rectangle, with the label assigned as “tomato”. Three keypoints were defined: P2, located at the junction of the calyx and the pedicel; P1, positioned 1 – 2 cm upward along the pedicel from P2; and P3, located at the bottom end of the tomato. An example of the annotated image is shown in **Figure 2**. If a keypoint is partially occluded, the suffix ‘hide’ is appended to its name; if a keypoint is invisible or absent, the suffix ‘missing’ is used instead. All annotations are stored in standard JSON format, including the image path, image dimensions (width, height, number of channels), as well as the bounding box of the tomato and the coordinates of each keypoint. The JSON annotation files were converted into TXT format for training purposes. The dataset was split into training and testing sets at a ratio of 8:2, resulting in 624 images for training and 156 images for testing. The overall image distribution conforms to a 4:1 ratio between the training set and the testing set. The visibility statistics of the annotated keypoints are summarized in **Table 1**.

**Table 1:** Instance in dataset.

Dataset	Image	Tomato	P0	P1	P2
Train	624	1934	890	800	4112
Val	156	451	273	174	906
All	780	2385	1163	974	5018

### 2.2 Methods

#### 2.2.1 YOLOv8-Pose

YOLOv8-Pose is based on the YOLOv8 neural network and introduces a keypoint localization module for human pose estimation on top of object detection. It is primarily used for human detection and pose estimation. Both YOLOv8 and YOLOv5 models were developed by the same author. The network consists of five main components: the input, Backbone, Neck, Head, and output. The Backbone continues the structure of previous versions, where an input image of 640×640 is processed to generate feature maps of sizes 80, 40, and 20, corresponding to downscaling factors of 8, 16, and 32, respectively. The C2f module is extensively used, which, compared to the C2 module in YOLOv5, introduces deeper convolutional feature fusion, providing richer gradient information. The Neck follows the structure of YOLOv5, utilizing a path aggregation network to enhance feature fusion. It achieves this by upsampling low-level features and fusing them with high-level features, as well as downsampling high-level features and merging them with low-level features. This improves the network’s ability to handle objects at different scales. The output layer includes both classification and detection. The coupled head is replaced with a decoupled head, where classification and regression tasks are handled by separate branches. This separation improves efficiency and accuracy when handling different tasks.

#### 2.2.2 Improved YOLOv8 - pose key - point recognition method for picking tomato fruits in the red - ripe stage

**Figure 3:**
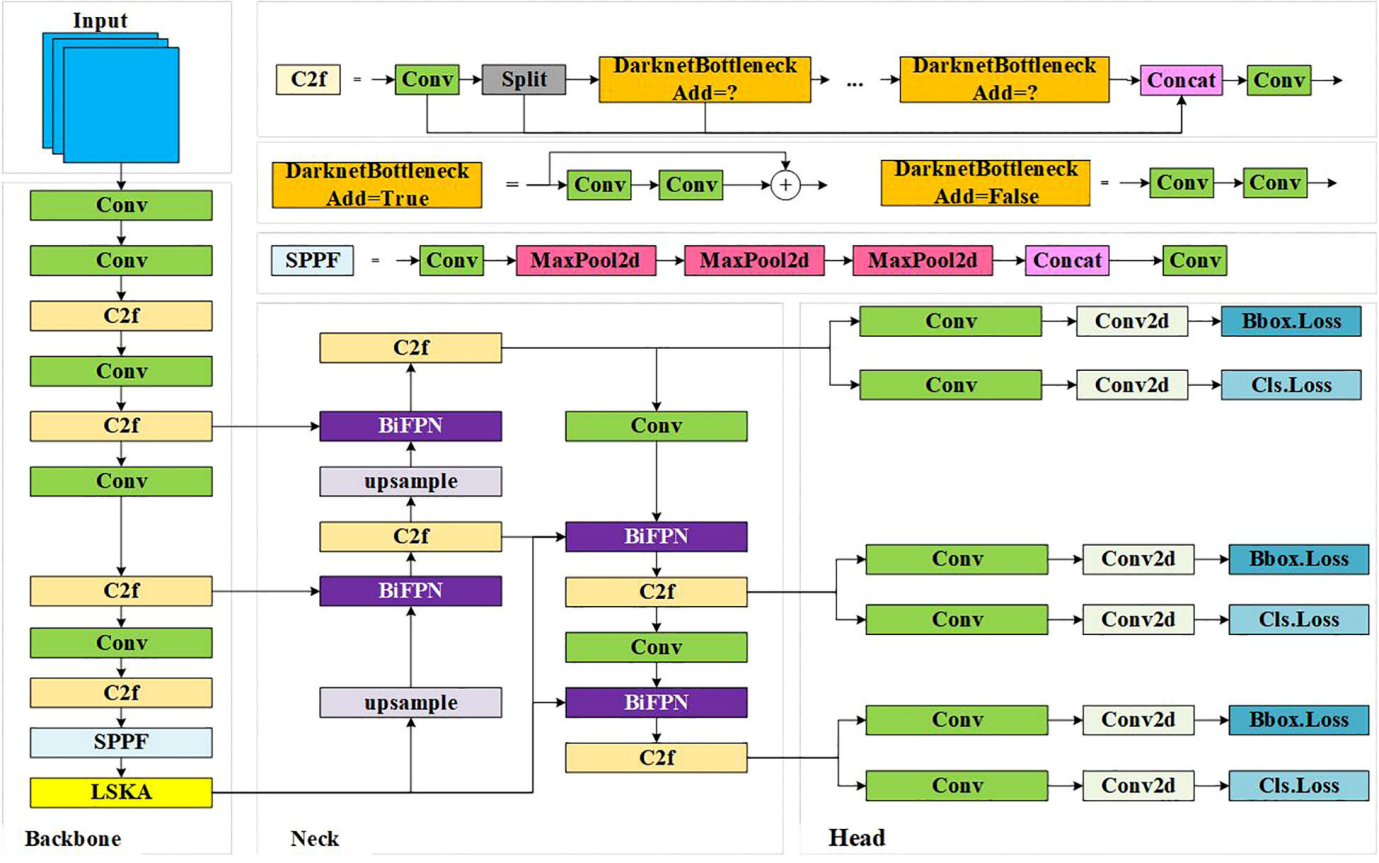
Structure of YOLOv8-LBP.

YOLOv8-Pose is a keypoint prediction model based on human pose estimation. Compared to human pose estimation, the number of keypoints, target categories, and feature characteristics of ripe tomatoes differ significantly. Therefore, the original model is not directly applicable to the recognition of ripe tomatoes and the detection of picking keypoints, requiring improvements to YOLOv8-Pose. To address challenges such as target occlusion, leaf occlusion, and mis-segmentation caused by the similar color of ripe tomatoes, this study designs and introduces a Large Separable Kernel Attention (LSKA) module to enhance the network’s ability to distinguish target features Deng et al. (2024). Additionally, a weighted Bi-directional Feature Pyramid Network (BiFPN) is used to replace the FPN structure in the Neck, allowing the network to learn the importance of different input features through learnable weights, thereby improving feature fusion capabilities. The overall framework of the improved YOLOv8-Pose algorithm is shown in **Figure 3**.

#### 2.2.3 BiFPN module

**Figure 4:**
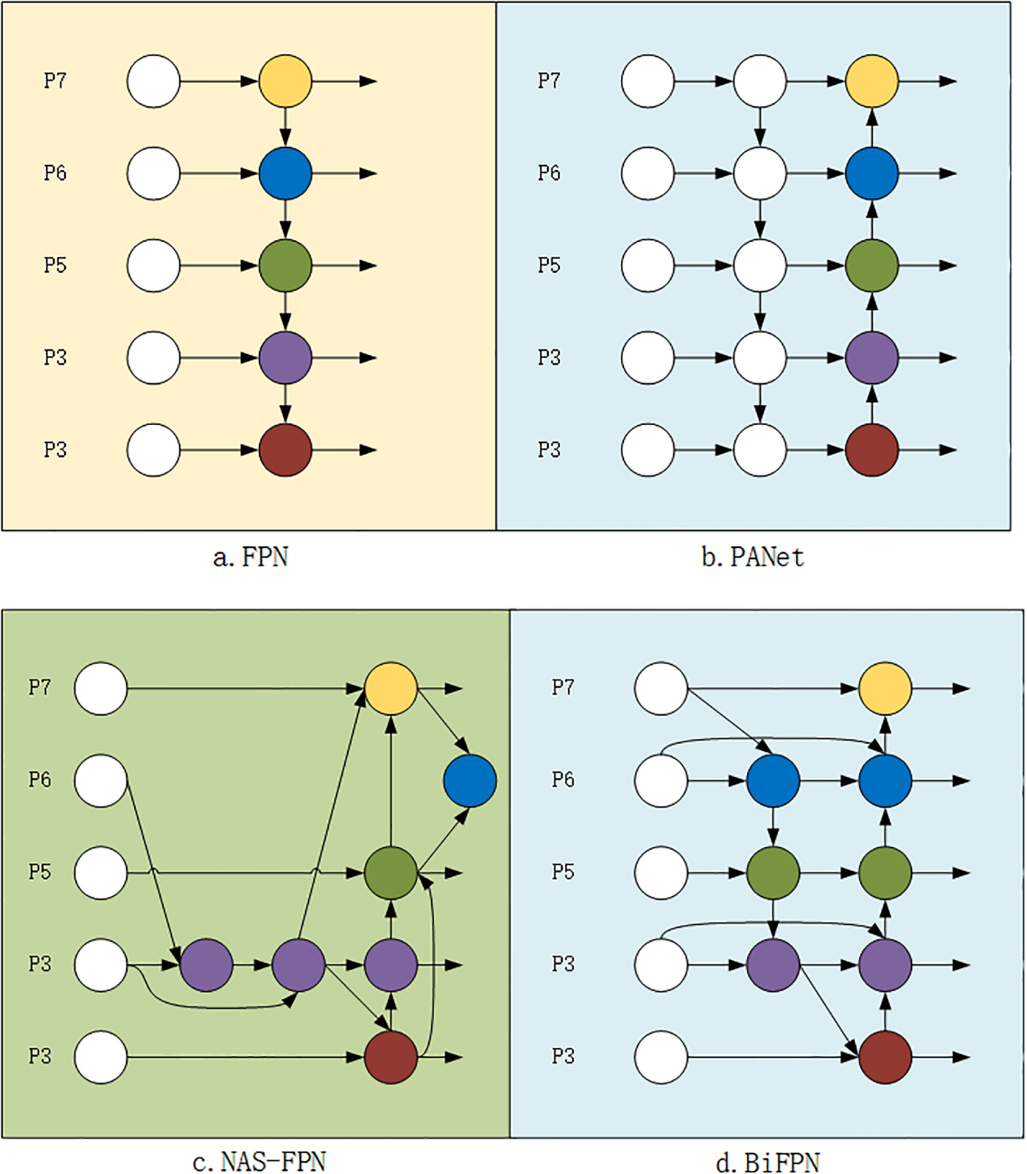
Feature network.

In object detection and keypoint estimation tasks, effectively acquiring and processing multi-scale feature information remains a major challenge. The traditional Feature Pyramid Network (FPN) (**Figure 4a**) Liu et al. (2018) aggregates multi-scale features in a top-down manner, which is often constrained by unidirectional information flow. To address this, the Path Aggregation Network (PANet) Tan et al. (2020) introduces an additional bottom-up path aggregation, as illustrated in **Figure 4b**. In the Neck of YOLOv8-Pose, the FPN structure from YOLOv5 is still adopted. High-level feature information is fused through the FPN+PAN structure, which improves the efficiency of feature transmission. However, this also increases computational complexity. Particularly when dealing with high-resolution inputs, it may result in high computational costs and reduced real-time performance. Moreover, the fixed architecture lacks adaptive adjustment capabilities for different tasks and datasets. To address the aforementioned issues such as unidirectional information flow, high accuracy at the cost of numerous parameters and heavy computation, as well as information loss and redundancy caused by simple feature concatenation, this paper proposes replacing the original FPN+PAN structure in YOLOv8-Pose with a BiFPN. Based on PANet and NAS-FPN (**Figure 4c**), BiFPN optimizes multi-scale feature fusion strategies. Its architecture is illustrated in **Figure 4d**.

Different feature input resolutions contribute differently to the final feature network output. Therefore, the network needs to learn the corresponding weights to balance feature importance. As shown in **Figure 4d**, feature maps from different levels are input into the BiFPN_Concat layer (taking BiFPN_Concat2 as an example). Given two feature maps $F_{0}\in R^{C_{0}\times H\times W}$ and $F_{1}\in R^{C_{1}\times H\times W}$ , where *C*_0_ and *C*_1_ denote the number of input channels, H and W represent the height and width of the feature maps respectively, the weights in the BiFPN_Concat2 module are initialized as w=[1,1], assigning equal initial importance to each input feature map. During each forward propagation step, weights are normalized to ensure stable training. To prevent division by zero, a small constant *ϵ* = 0.0001 is used. The formula for weight normalization is as follows (Equation 1):


(1) $$weight=\frac{w}{\sum_{i=0}^{n}w_{i}+\epsilon }$$

In BiFPN_Concat2, the number of input feature maps is n = 2. After normalization, the weights are represented as weight = [ $weight_{0}$ , $weight_{1}$ ], where $weight_{0}$ + $weight_{1}$ = 1. Each input feature map is multiplied by its corresponding normalized weight: $F_{0}^{weighted}=weight_{\;0}\times F_{\;0}$ , $F_{1}^{weighted}=weight_{\;1}\times F_{\;1}$ During training, the model optimizes w based on loss feedback, which in turn updates weight values dynamically. Finally, BiFPN_Concat2 concatenates the weighted feature maps along the channel dimension to obtain the output feature map (Equation 2):


(2) $$F_{out}=concat\left(F_{0}^{weighted},F_{1}^{weighted}\right)$$

At this stage, the output feature map is represented as $F_{out}=R^{\left(C_{0}+C_{1}\right)\times H\times W}$ , successfully fusing feature information from *F*_0_ and *F*_1_.

#### 2.2.4 LSKA attention mechanism

To address the challenges of occlusion among small targets and mis-segmentation caused by similar colors between ripe tomato fruits, this paper designs and incorporates a Large Separable Kernel Attention (LSKA) module to enhance the network’s ability to distinguish target features Deng et al. (2024). By enlarging the receptive field of the convolutional kernels, the module focuses more on the overall shape characteristics of the target, thereby reducing the interference from background textures and color similarity in the detection results Ding et al. (2022). As the kernel size increases, LSKA can effectively capture broader contextual information, thus improving the recognition performance of small targets in complex scenes. However, directly increasing the kernel size also significantly raises the computational cost and memory usage, posing challenges to the model’s efficiency. LSKA decomposes a *k* × *k* depthwise convolution (DW-Conv) into two sequential 1D DW-Convs with kernel sizes of 1×(2*d* −1) and (2*d* −1)×1, as well as two sequential 1D depthwise-dilated convolutions (DW-D-Conv) with kernel sizes of $\left\lfloor \frac{k}{d}\right\rfloor \;\times \;1$ and $1\times \left\lfloor \frac{k}{d}\right\rfloor$ . The outputs are then passed through a 1×1 convolution. The depthwise-dilated convolutions are responsible for capturing global spatial information from the depthwise convolution output. This design achieves an effective receptive field equivalent to that of a large kernel while avoiding the quadratic increase in computational cost typically caused by using large kernels in depthwise convolutions alone. Given an input feature map *F*_0_ ∈ *R* ^C×*H*×W^, where C is the number of input channels and H and W represent the height and width of the feature map, the output of LSKA is computed as follows (Equations 3–6):


(3) $$Z^{C}=\sum_{H,W}W_{\left(2d-1\right)\times 1}^{C}\;*\;\left(\sum_{H,W}W_{1\times \left(2d-1\right)}^{C}\;*\;F^{C}\right)$$

(4) $$Z^{C}=\sum_{H,W}W_{\left\lfloor \frac{k}{d}\right\rfloor \times 1}^{C}\;*\;\left(\sum_{H,W}W_{1\times \left\lfloor \frac{k}{d}\right\rfloor }^{C}\;*\;\bar{Z^{C}}\right)$$

(5) $$A^{C}=W_{1\times 1}\;*\;Z^{C}$$

(6) $$F^{C}=A^{C}\;⊗\;F^{C}$$

Where d is the dilation rate and k represents the maximum receptive field. * and ⊗ denote convolution and Hadamard product (element-wise multiplication), respectively. $\bar{Z^{C}}$ represents the output of two cascaded 1D depthwise convolutions (DW-Conv) with kernel sizes of 1×(2d−1) and (2d−1)×1 , respectively. $Z^{C}$ represents the output of two cascaded 1D depthwise dilated convolutions (DW-D-Conv) with kernel sizes of $\left\lfloor \frac{k}{d}\right\rfloor \times 1$ and $1\times \left\lfloor \frac{k}{d}\right\rfloor$ , respectively (where ⌊.⌋ denotes the floor function). $A^{C}$ represents the output of applying a 1 × 1 convolution to $Z^{C}$ . $\bar{F^{C}}$ represents the result of the Hadamard product between the feature map $A^{C}$ and the input feature map $F^{C}$

## 3 Model training

### 3.1 Training environment and parameter settings

The main hardware configuration for training and testing is shown in **Table 2**. [] The training epoch is set to 100, batch size is 32, input image size is 640×640 pixels, the initial learning rate is set to 0.001, momentum factor is 0.937, and other parameters are kept at default.

**Table 2:** Experimental environment.

Configuration	Parameter
CPU (Central Processing Unit)	Intel (R) Xeon (R) Gold 6240 CPU @ 2.60GHz
GPU (Graphics Processing Unit)	NVIDIA GeForce RTX 2080Ti
Video Memory Capacity/MB	11264
Training Environment	CUDA 11.3, cuDNN 8.2
Operating System	Ubuntu 20.04 LTS
Development Environment	python 3.11.5, Pytorch 2.1.0

### 3.2 Evaluation metrics

To validate the effectiveness of the proposed model in detecting tomatoes, commonly used quantitative metrics in the field of object detection were employed, including precision (*P*), mean average precision at Intersection over Union (IoU) threshold 50% (*mAP*_50_), and mean average precision across IoU thresholds from 50% to 95% (*mAP*_50−95_), to comparatively analyze the tomato fruit detection performance. Specifically, *mAP*_50_ refers to the mean average precision calculated at an Intersection over Union (IoU) threshold of 50%, while *mAP*_50−95_ represents the mean average precision calculated across IoU thresholds ranging from 50% to 95%, averaged over multiple thresholds. The detailed formulas are as follows (Equation 7):


(7) $$p=\frac{TP}{TP+FP}$$

In the formula: *TP* is the number of true positive detections (correctly detected boxes). *FP* is the number of false positive detections (incorrectly detected boxes). *FN* is the number of false negative detections (missed targets). *AP* is the Average Precision for a specific class, and *mAP* is the mean Average Precision across all detection classes. The specific calculation formulas are as follows (Equations 8, 9):


(8) $$AP=\int_{0}^{1}P\left(R\right)dR$$

(9) $$mAP=\frac{1}{N}\sum_{1}^{N}AP_{i}$$

In the formula: *N* is the number of object categories to be detected. In this experiment, only the red-ripe tomato category needs to be detected, so *N* = 1. The target keypoint similarity *OKS_p_* is obtained through detection. From *OKS_p_* Maji et al. (2022), the Average Precision *AP* can be derived, and from AP, the *mAP_kp_* is calculated as the evaluation metric for keypoints. The formula for calculating *OKS_p_* is (Equation 10):


(10) $$OKS_{p}=\frac{\sum_{i}\exp\;(\frac{-d_{pi}^{2}}{2s_{p}^{2}\delta_{i}^{2}})\delta }{\sum_{i}pi}(v_{pi}>0)$$

In the formula:

• $d_{pi}$ is the Euclidean distance between the predicted and ground truth values of the i-th keypoint of the p-th target.

• $v_{pi}\;$ is the visibility index of the i-th keypoint of the p-th target.

• *v_i_* is the area of the bounding box of the p-th target.

• $\delta_{i}$ is the standard deviation between the label and the actual value of the i-th keypoint.

The formula for calculating *AP* is (Equation 11):


(11) $$AP=\frac{\sum_{m}\sum_{p}\beta }{\sum_{m}\sum_{p}1}$$

In the formula (Equation 12):


(12) $$\beta =\{\begin{array}{ll} 0 & \;OKS_{p}\leq T\\ OKS_{p} & \;OKS_{p}>T \end{array}$$

In the formula, *T* represents the threshold, ranging from 0.5 to 0.95.

### 3.3 Model results and analysis

To comprehensively evaluate the overall performance of the improved YOLOv8-Pose network model, joint training and validation were conducted on a custom ripened-tomato dataset.

#### 3.3.1 Experimental results on the custom dataset

The LSKA attention mechanism decomposes large convolutional kernels to achieve a larger receptive field, enhancing the model’s feature extraction capability. We set kernel sizes of 7, 11, 23, 35, 41, and 53, and trained the YOLOv8-LBP model on a self-collected red-ripe tomato dataset. When the kernel size was 23, the model achieved the highest Precision of 0.944; at kernel size 7, the *mAP*_50_ peaked at 0.926 with the smallest parameter count of 3.164M; kernel size 41 yielded the best *mAP*_50−95_ and keypoint *mAP*_50−95_ − *kp* scores of 0.71 and 0.785, outperforming other sizes; while kernel size 53 resulted in the highest FPS of 107.53. Considering the balance between detection performance and computational efficiency, we selected the LSKA attention mechanism with kernel size 41 for subsequent experiments. Details are shown in **Table 3**.

**Table 3:** Impact of different LSKA kernel sizes on YOLOv8-LBP detection performance and efficiency.

Kernel size	P	mAP_50_	mAP_50−95_	mAP_50−95_ − *kp*	Params (M)	FPS
7	0.924	0.926	0.689	0.763	3.164	97.01
11	0.918	0.904	0.690	0.742	3.169	98.04
23	0.944	0.907	0.678	0.762	3.172	90.10
35	0.927	0.903	0.679	0.749	3.174	85.47
41	0.927	0.917	0.710	0.785	3.175	99.01
53	0.925	0.915	0.689	0.759	3.177	107.53

#### 3.3.2 Experimental results on the self-constructed ripe tomato dataset

**Figure 5:**
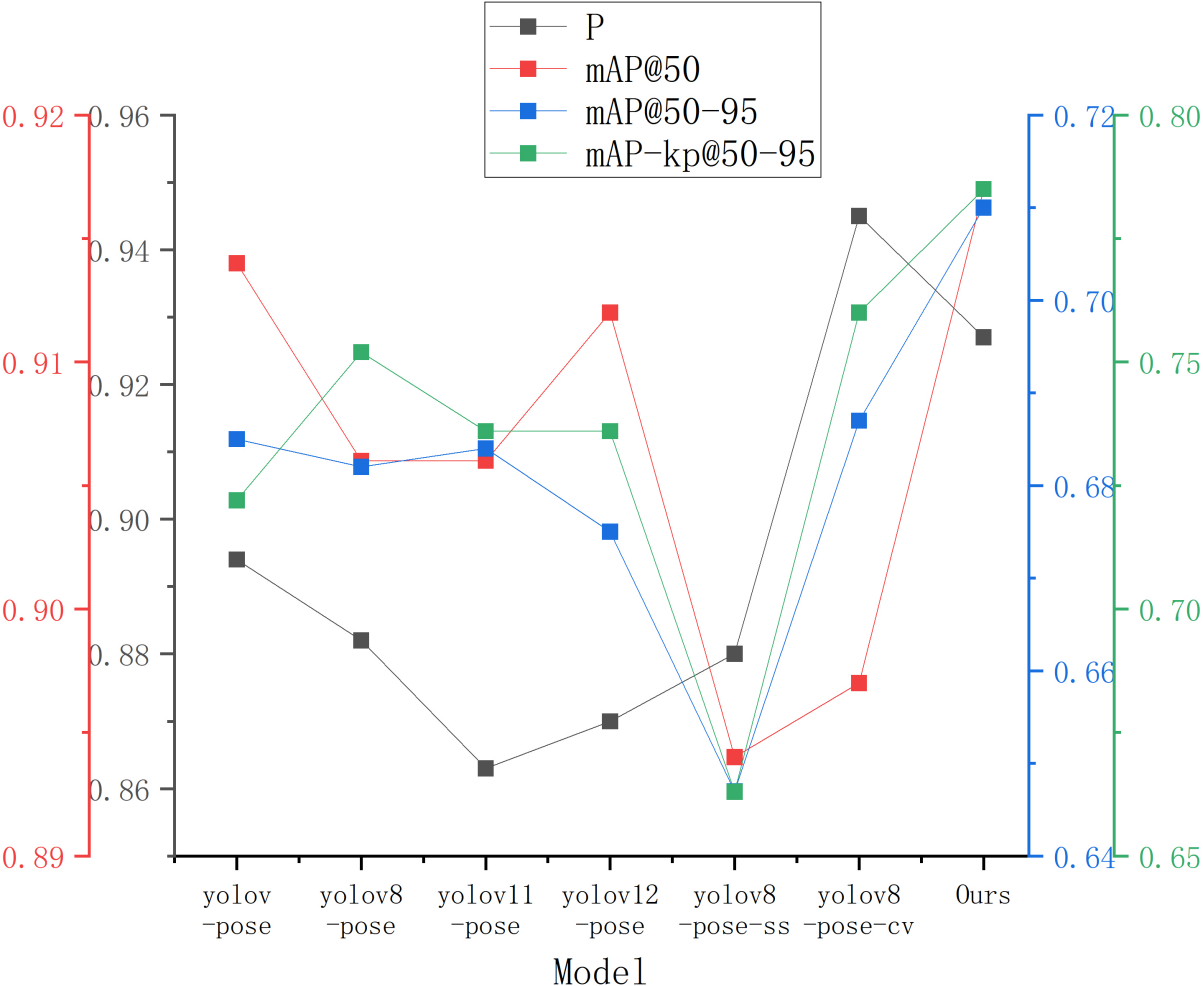
Results of different models on self-made red-ripe stage tomato dataset.

In the experiments on object recognition and keypoint prediction, we evaluated the performance of several models including YOLOv5-Pose, YOLOv8-Pose, YOLOv11-Pose, YOLOv12-Pose, YOLOv8-Pose-ss Guo et al. (2024), YOLOv8-Pose-cv Mochen et al. (2023), and YOLOv8-LBP on a self-collected red-ripe tomato dataset. The results are presented in **Table 4**. Comparative analysis shows that YOLOv8-LBP achieved 92.7%, 91.7%, 71.%, and 78.5% on Precision, *mAP*_50_, *mAP*_50−95_, and *mAP*_50−95_ − *kp* metrics, respectively. Although its Precision is slightly lower than YOLOv8-Pose-cv’s 0.945, it surpasses all other models in the remaining metrics, demonstrating its outstanding performance in red-ripe tomato detection and harvesting keypoint localization tasks. **Figure 5** illustrate the experimental performance and comparative results of different models across various metrics.

**Table 4:** Results of different models on self-made red-ripe stage tomato dataset.

model	*P*	*mAP*_50_	*mAP*_50-95_	*mAP*_50−95_ − *kp*
YOLOv5-Pose	0.874	0.904	0.675	0.722
YOLOv8-Pose	0.882	0.906	0.682	0.752
YOLOv11-Pose	0.863	0.906	0.684	0.736
YOLOv12-Pose	0.87	0.912	0.675	0.736
yolov8-pose-ss	0.88	0.894	0.647	0.663
yolov8-pose-cv	0.945	0.897	0.687	0.76
Ours	0.927	0.917	0.71	0.785

**Figure 6:**
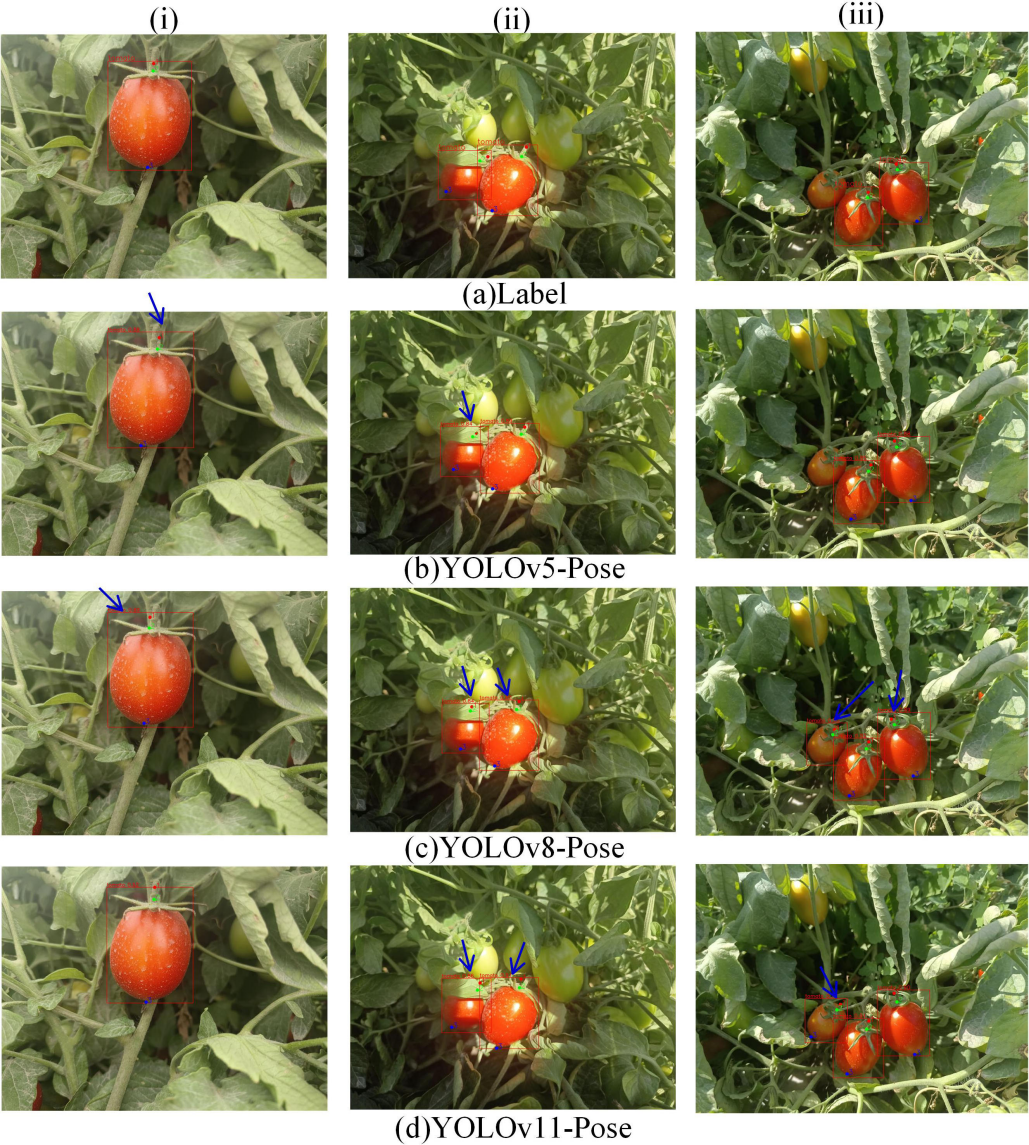
Examples of different experimental results.

**Figure 7:**
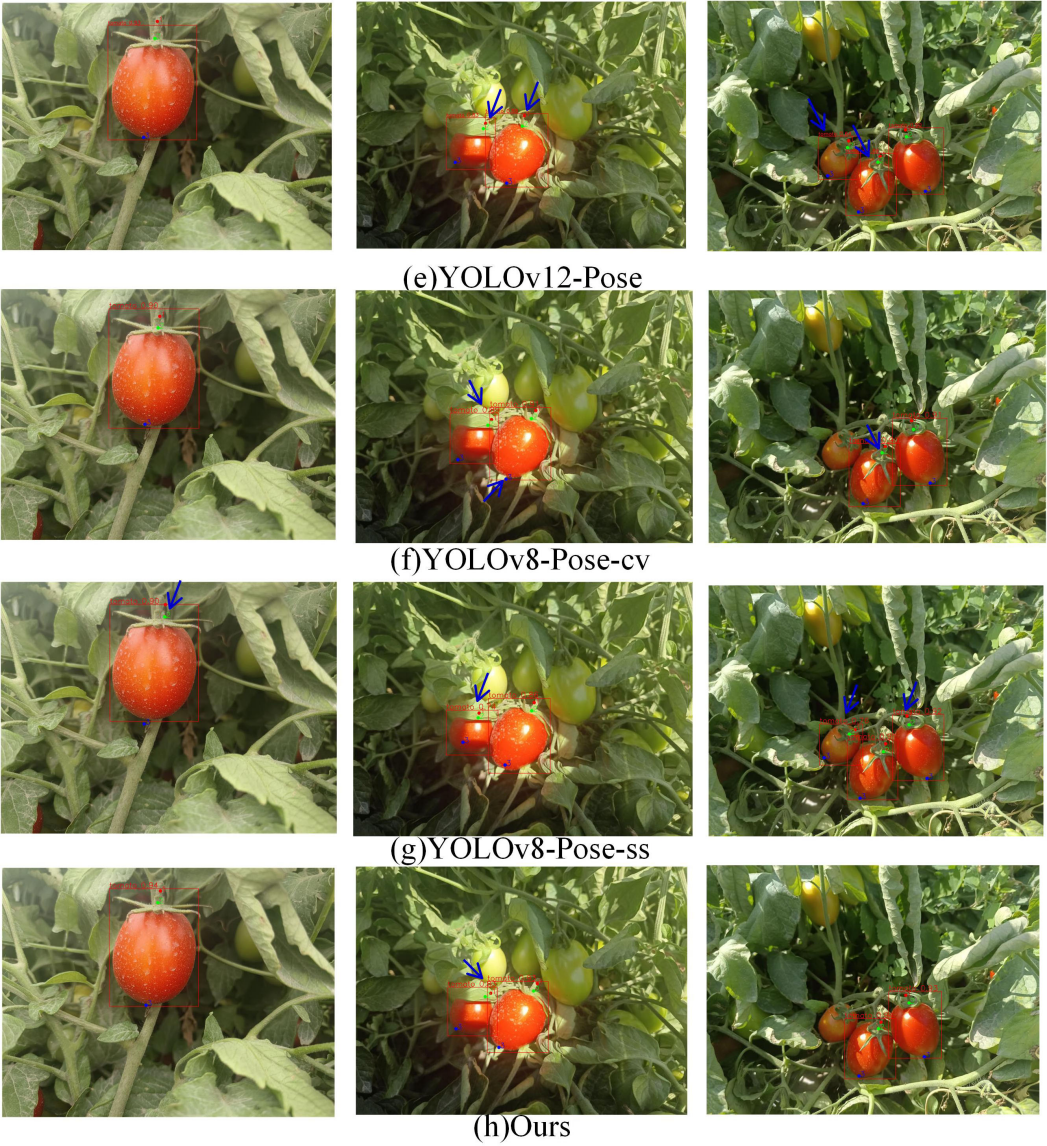
Examples of different experimental results.

The experimental results comparing YOLOv5-Pose, YOLOv8-Pose, YOLOv11-Pose, YOLOv12-Pose, YOLOv8-Pose-ss, YOLOv8-Pose-cv, and YOLOv8-LBP models for ripe tomato recognition and picking keypoint detection are shown in **Figures 6**, **7**. From column (i), it can be seen that models b and c have certain deviations in keypoint prediction, while models d, e, f, and g have lower tomato recognition confidence compared to model h; Column (ii) shows that under leaf occlusion, models c, d, e, and f exhibit multiple slight deviations in keypoint prediction, models b and g show larger deviations, while model f, despite some minor deviations, achieves better overall keypoint prediction accuracy than other models; Column (iii) indicates that models c, d, e, and g have false positives in tomato recognition, models c, d, e, f, and g all show certain deviations in keypoint prediction, with model f having the most accurate keypoint predictions and the highest tomato recognition confidence.

**Figure 8:**
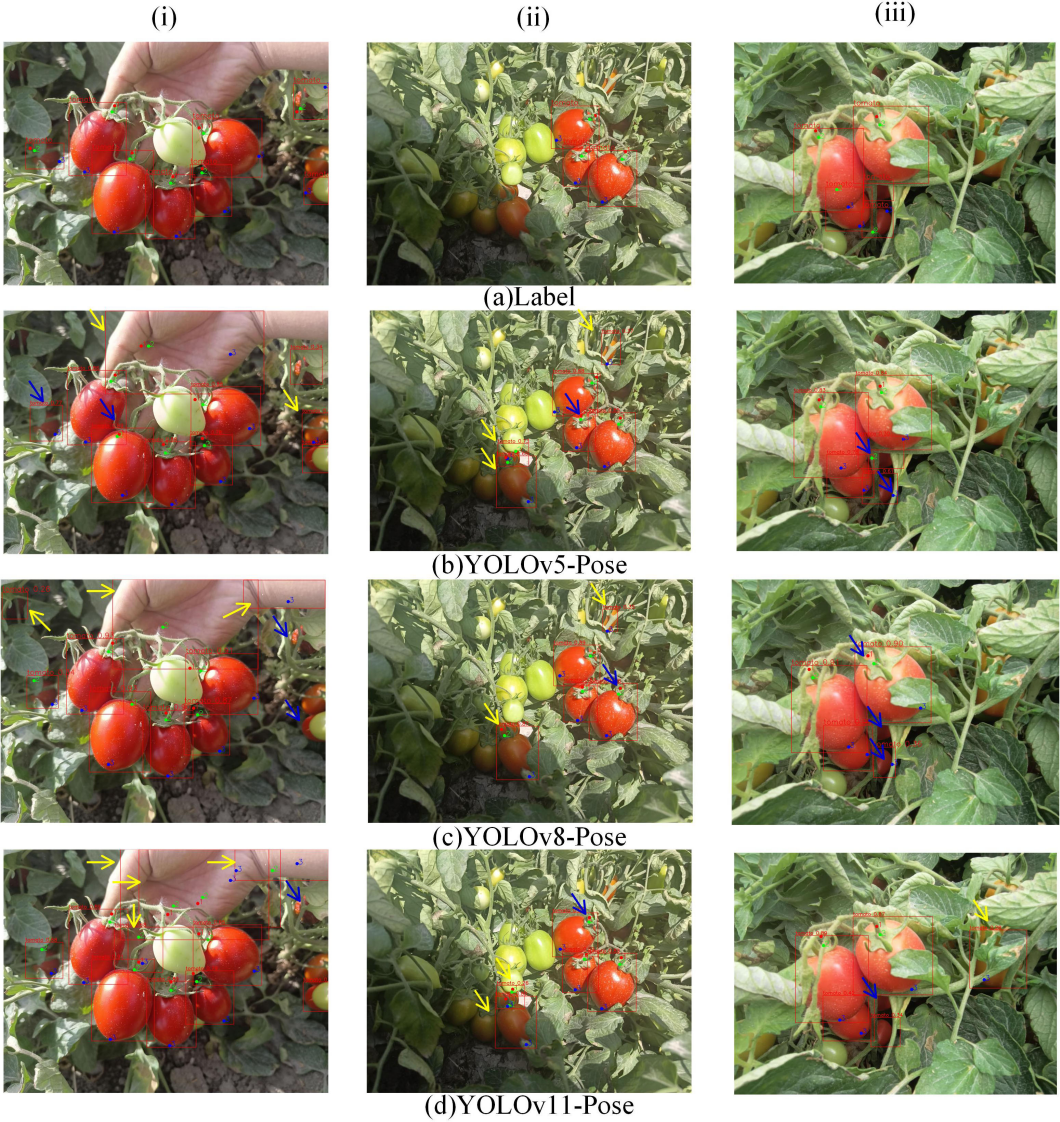
Comparative analysis of detection and prediction results of various models in complex natural scenes.

**Figure 9:**
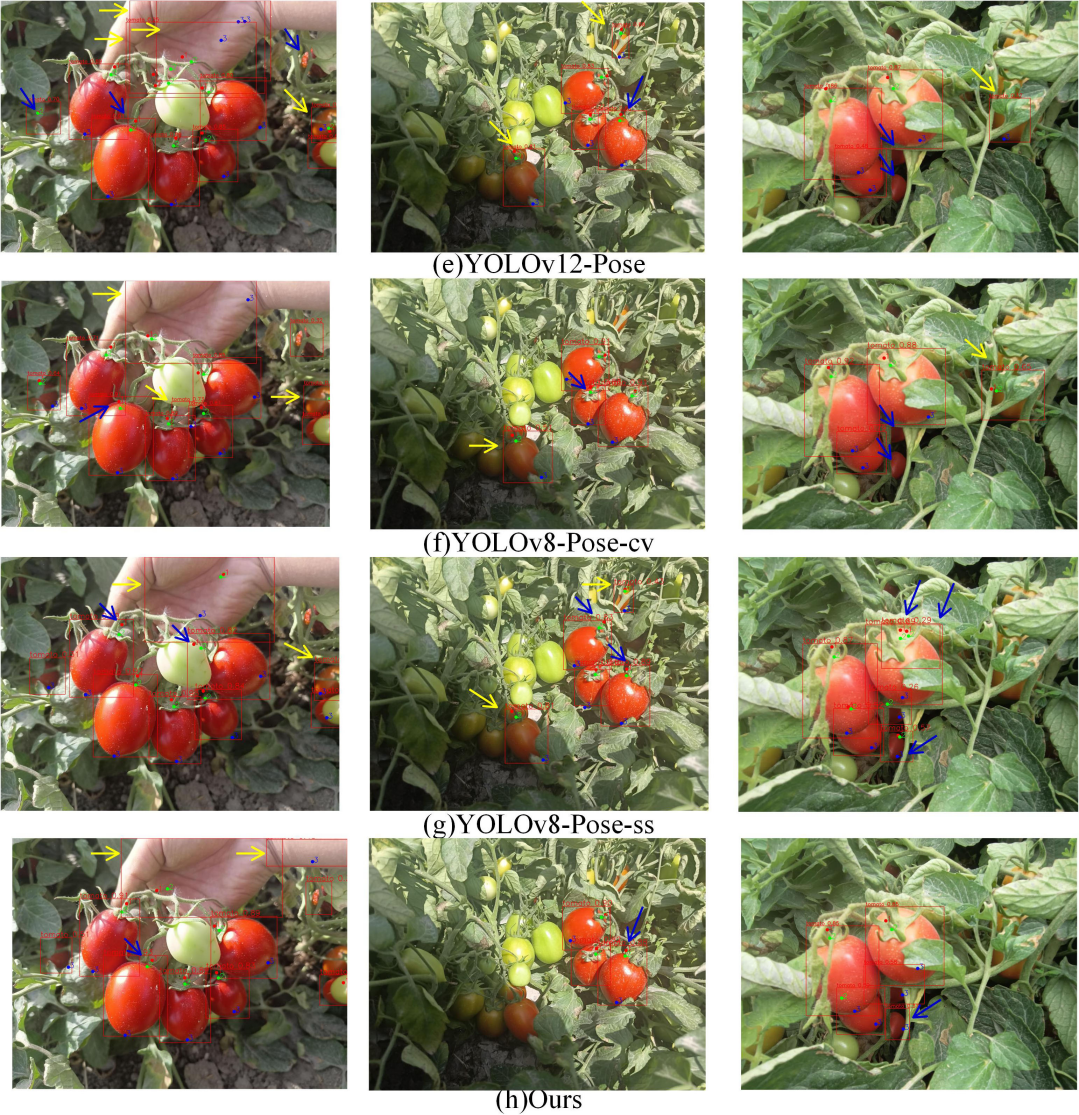
Comparative analysis of detection and prediction results of various models in complex natural scenes.

**Figures 8**, **9** present a comparison of different models’ results on object recognition and keypoint detection in complex natural environments. Column (i) includes cases with hand background interference, clusters of tomatoes, and environmental occlusions; column (ii) shows scenarios with strong direct sunlight; column (iii) shows tomato clusters and fruit occlusions. Yellow arrows indicate false positives, while blue arrows denote other types of errors. In column (i), models c, d, e, and f exhibit three or more false positives; models b, g, and h have two false positives; models c, d, e, and g show missed detections of tomatoes; models b, f, and h show no missed detections; models b, d, e, and g have large deviations in keypoint predictions within multiple bounding boxes; models c, f, and h show large deviations within a single bounding box. In column (ii), models b, c, d, e, f, and g all have false positives under shadow conditions; models b, c, e, and g have false positives under direct sunlight; model g has two bounding boxes with large keypoint prediction deviations, while the other models have one such deviation each; model h has no false positives. In column (iii), models d, e, and f have false positives; models c, d, e, and f have missed detections; model g shows repeated detection of tomatoes; model h accurately recognizes every ripe tomato; models b, c, g, and h have large keypoint prediction deviations. Overall, model h outperforms the others. YOLOv8-LBP effectively addresses missegmentation caused by similar tomato fruit colors and occlusions in natural environments, providing more accurate keypoint localization, making it suitable for robotic harvesting applications.

To comprehensively evaluate the performance of different models, three commonly used computational metrics were selected for comparative analysis: the number of parameters (Params), Giga Floating-point Operations per Second (GFLOPs), and frames per second (FPS). The number of parameters (Params) represents the total count of all trainable parameters in the model, reflecting its complexity and storage requirements. GFLOPs measure the computational cost required for a single forward inference. FPS indicates how many image frames the model can process per second during actual operation and is an important metric for evaluating real-time performance. **Table 5** presents a comparison of these three computational metrics across different models. As shown in **Table 5**, YOLOv8-Pose-ss has the smallest number of parameters, the highest FPS, and the lowest computational cost (GFLOPs), indicating that our proposed model does not have an advantage in computational efficiency. Compared with the baseline model, our approach slightly increases the number of parameters (from 3.08M to 3.175M), raises GFLOPs by 0.1, and marginally improves FPS (from 96.15 to 99.01). Although computational cost increases slightly, this overhead is reasonable and acceptable, especially given the substantial improvements in accuracy: relative to the baseline model, object detection Precision improves by 4.5%, *mAP*_50_ by 1.1%, *mAP*_50−95_ by 2.8%, and keypoint detection accuracy (*mAP*_50−95_ − *kp*) by 3.3%. Compared with YOLOv8-Pose-ss, our model achieves 4.7% higher Precision (0.927 vs. 0.880), 2.3% higher *mAP*_50_ (0.917 vs. 0.894), 6.3% higher *mAP*_50−95_ (0.710 vs. 0.647), and 12.2% higher *mAP*_50−95_ − *kp* (0.785 vs. 0.663). These results indicate that our approach achieves a favorable trade-off between accuracy and computational cost. Furthermore, with the continuous advancement of embedded device computing power and the widespread adoption of cloud computing, the impact of a slight increase in computational resources on practical deployment is gradually diminishing. Therefore, we believe the proposed method retains strong practical applicability and scalability while maintaining high accuracy.

**Table 5:** Performance comparison of different models under typical evaluation metrics.

Model	Parameters (M)	FPS	GFLOPs
YOLOv5-Pose	2.58	75.76	7.3
YOLOv8-Pose	3.08	96.15	8.4
YOLOv11-Pose	2.65	84.75	6.6
YOLOv12-Pose	2.63	73.5	6.6
YOLOv8-Pose-ss	1.91	113.64	5.3
YOLOv8-Pose-cv	5.55	109.9	22.7
Ours	3.175	99.01	8.5

#### 3.3.3 Ablation experiments

To verify the effectiveness of each module, an ablation study was conducted based on the YOLOv8n-Pose model by gradually adding and replacing components. The results are shown in **Table 6**. Compared to the original YOLOv8n-Pose, adding the LSKA attention mechanism alone expanded the model’s effective receptive field, enabling the extraction of richer feature information. This led to increases of 0.7% and 0.5% in *mAP*_50_ and *mAP*_50−95_, respectively. When adding the BiFPN module alone, the model’s ability to process multi-scale feature information was enhanced, resulting in a 1.9% improvement in precision (*P*). When both LSKA and BiFPN were added simultaneously, the model not only obtained richer feature information but also leveraged BiFPN’s ability to adaptively adjust the importance of these features, achieving more effective feature fusion. This allowed the model to better focus on the overall shape features of the target while reducing the interference of background textures and color similarity, leading to improvements of 4.5%, 1.1%, 2.8%, and 3.3% in *P*, *mAP*_50_, *mAP*_50−95_, and *mAP*_50−95_−*kp*, respectively. The ablation experiments demonstrate that the two improvements proposed in this study are effective.

**Table 6:** Ablation experiment results.

LSKA	BiFPN	*P*	*mAP*_50_	*mAP*_50-95_	*mAP*_50−95_ − *kp*
×	×	0.882	0.906	0.682	0.752
✓	×	0.881	0.913	0.687	0.748
×	✓	0.901	0.899	0.679	0.74
✓	✓	0.927	0.917	0.71	0.785

## 4 Conclusion

To achieve accurate detection of harvesting keypoints for ripe tomatoes in complex scenes, this paper draws on human pose estimation methods and proposes the YOLOv8-LBP model based on BiFPN and LSKA attention mechanisms to accomplish the recognition of ripe tomato fruits and the prediction of their harvesting keypoints. The weighted bidirectional feature pyramid network (BiFPN) is used to learn different input features, while the LSKA attention mechanism enlarges the model’s effective receptive field, enabling more efficient multi-scale target information extraction and suppressing the influence of interfering features. Ablation experiments show that on the self-made ripe tomato dataset, the YOLOv8-LBP model improves *P*, *mAP*_50_, *mAP*_50−95_, and *mAP*_50−95_ − *kp* by 4.5%, 1.1%, 2.8%, and 3.3% respectively, demonstrating significant enhancement; Only a small computational overhead was introduced, with the number of parameters increasing by 0.095 M, GFLOPs increasing by 0.1, and inference speed improving by 2.86 FPS. Experimental results confirm the effectiveness of the proposed improvements. The research outcomes provide effective support for enhancing ripe tomato harvesting capabilities in production and hold positive significance for the future development of smart agriculture.

## Data Availability

The raw data supporting the conclusions of this article will be made available by the authors, without undue reservation.
